# Reconfigurable broadband metasurfaces with nearly perfect absorption and high efficiency polarization conversion in THz range

**DOI:** 10.1038/s41598-022-23536-8

**Published:** 2022-11-05

**Authors:** Thi Minh Nguyen, Dinh Lam Vu, Thi Quynh Hoa Nguyen, Jung-Mu Kim

**Affiliations:** 1 Vietnam Academy of Science and Technology, Graduate University of Science and Technology, 18 Hoang Quoc Viet, Cau Giay, Hanoi, 10000 Vietnam; 2 School of Engineering and Technology, Vinh University, 182 Le Duan, Vinh, Nghe An 43000 Vietnam; 3 Department of Electronic Engineering, Jeonbuk National University, Jeonju, 54896 Republic of Korea

**Keywords:** Metamaterials, Terahertz optics

## Abstract

Reconfigurable metasurfaces (RMSs) that enable the switching function of absorption and polarization conversion have attracted increasing attention. However, the design of RMSs to achieve wideband and high efficiency for both absorption and polarization conversion functions simultaneously remains a great challenge. Here, we propose the design of a RMS structure with a high-efficiency cross-polarization conversion and nearly perfect absorption. The reconfiguration between different functions of polarization conversion and absorption is obtained based on the reversible insulator-to-metal phase transition of Vanadium dioxide (VO$_{2}$). When the VO$_{2}$ is in insulator state, the RMS realizes the cross-polarization conversion function in the wideband of 1.04–3.75 THz with a relative bandwidth up to 113 $\%$ due to the multi-resonant modes of electric and magnetic resonances. Meanwhile, the nearly-perfect absorption is achieved in the range of 1.36–3.38 THz with the corresponding relative bandwidth up to 85 $\%$ for the VO$_{2}$ in metallic state. Specially, the wideband and high-efficiency performance of these functionalities is maintained for a wide angle incidence. The capability of bi-functional switch and integration with polarization conversion and absorption in a single metasurface structure endowed with both wideband and high-efficiency characteristics for a wide incident angle is very promising for emerging RMS devices in the terahertz region.

## Introduction

Metamaterials have enabled the realization of numerous phenomena and functionalities that is not found in naturally occurring materials^1–3^. However, owing to their 3D-form with bulky volume, metamaterials show the disadvantages of manufacturing complexity, high loss, and strong dispersion^4,5^, this limits the practical applications of metamaterials. To overcome these drawbacks of the metamaterials, metasurfaces, a two-dimensional (2D) or planar version of metamaterials with subwavelength thickness, have been proposed for various applications due to their advantages of low profile, low loss, and easy fabrication. Furthermore, metasurfaces have exhibited the capability of manipulating electromagnetic (EM) waves in microwave and optical frequencies. Such as, designed metasurfaces have demonstrated a variety of unique phenomena and fascinating applications such as beam-steering^6,7^, flat lens^8^, optical holograms^9^, broadband absorber^10,11^, polarization converter^12,13^, and polarimeters^14^.

Generally, conventional metasurfaces have been usually designed for a single functionality. Recently, reconfigurable metasurfaces (RMSs) have been proposed that exhibit diversified functionalities together into one single device including bi-functionalities of linear polarization conversion (LPC) and circular polarization conversion (CPC)^15–20^, absorption (ABS) and reflection^5,21–24^, ABS and polarization conversion (PC)^25–38^. To achieve reconfigurable multifunctional metasurfaces, some approaches have been proposed such as mechanical shape-changing^31,39^ and integrating with lumped elements^5,28–30,40^. However, these approaches process some drawbacks such as the limitation of the working band and sophistication of the fabrication process. In addition, most of these studies have successfully designed microwave multifunctional metasurfaces. Therefore, metasurfaces that facilitate the effective integration of multiple functionalities into one structure have become an emerging research area, especially interesting for the terahertz (THz) range^41^. More recently, the incorporation of standard metasurfaces with phase-change materials (PCMs), such as Chalcogenide GeSbTe(GST) alloys^42^, Vanadium dioxide (VO$_{2}$)^23,33–38^ and graphene^18,20,26,27,43^ have been proposed to realize the multifunctional metasurfaces. However, compared with PCM materials, graphene based method is still required high cost and sophistication for the manufacturing process. Among these PCM materials, VO$_{2}$, as a phase transition material, has advantages like a fast response, large modulation depth, and multiple modulation methods such as optical pumping, thermal control, and extra electric fields^44^. Furthermore, the drastic variation of optical and electrical properties of VO$_{2}$ is realized during the phase transition, this is due to the transformation of structural properties from an insulation phase (low temperature) to a metallic phase (high temperature) at around 68°^45,46^. Therefore, VO$_{2}$ materials are widely used in THz active RMSs. Despite significant advances in PCMs integrated metasurfaces with active reconfigurable configuration, the design of metasurface structures that can be actively reconfigured the distinct functionalities over a wide frequency band along with high efficiency remains a huge challenge and a largely unexplored research area to date^21,41^.

In this study, we design a wide-angle insensitive and reconfigurable wideband metasurface with two functionalities operating at THz range based on VO$_{2}$, which undergoes the insulator-metal transition. Our simulations demonstrate that the designed metasurface can be switched from a broadband absorber to a reflective broadband cross-polarization converter by varying the insulator-to-metal transition in VO$_{2}$. When the VO$_{2}$ is in the metallic state, the metasurface would efficiently absorb normally incident THz waves in the range from 1.36 to 3.38 THz with the total ABS exceeding 90$\%$ under both transverse electric (TE) and transverse magnetic (TM) polarizations. Once VO$_{2}$ is in its insulator state, the metasurface becomes a broadband reflective cross polarization converter with the LPC efficiency exceeding 90$\%$ within the relative bandwidth (RBW) of 113$\%$. Besides the excellent performance at normal incidence, the designed metasurface reveals that the broadband and high-efficiency performance of both ABS and LPC is maintained for a wide incidence angle. Furthermore, the physical mechanism of RMS with ABS and PC has been thoroughly numerically investigated.

## Structure design and method

**Figure 1:**
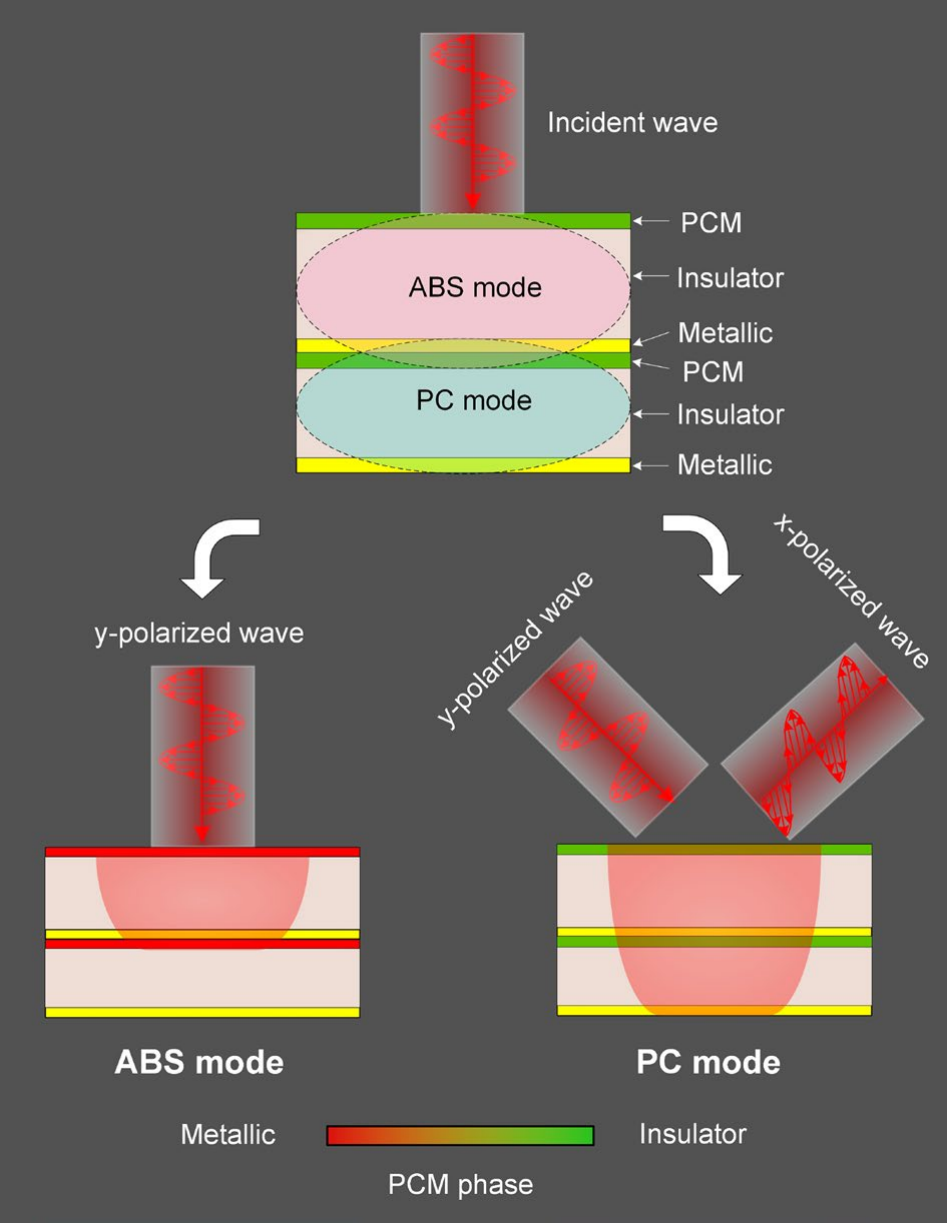
Design approach for RMSs with ABS and PC functions based on the reversible metallic-to-insulator phase transition of PCM.

To obtain the RMS that enables switching between ABS and PC modes, we proposed the RMS structure that combines two stacks of metasurfaces as shown in Fig. 1. One stack works in ABS mode when PCM material is in metallic state that combines a metallic PCM resonator and a metallic PCM layer as a ground plane separated by an insulator layer. Other stack works in PC mode when PCM material is in an insulator state that combines a metallic resonator and a metallic layer as a ground plane sandwiched by the PCM and insulator layers. It should be noticed that the phase transition of the PCM layer embedded between both insulator layers plays an important role in switching the functionality modes from ABS mode to PC mode by blocking to transmitting the incident EM wave, respectively. Meanwhile, the PCM resonator is used to enlarge the ABS bandwidth due to the PCM is a highly lossy metallic despite being in fully metallic state^41^.

**Figure 2:**
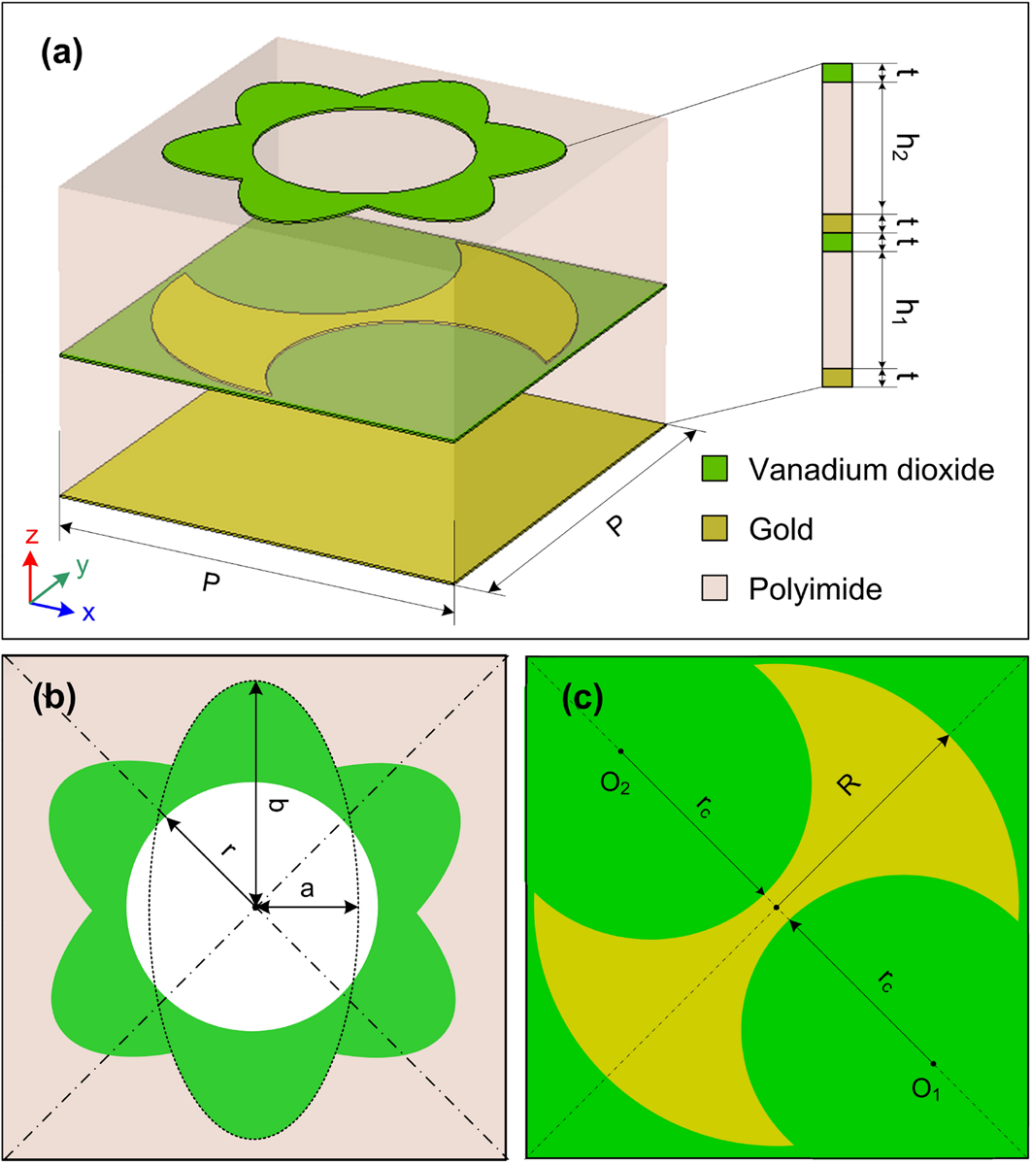
The proposed RMS: (**a**) 3D-model, (**b**) top-view of resonant patch for ABS mode, and (**c**) top-view of resonant patch for PC mode.

This designed approach has realized some RMS structures using VO$_{2}$ and Gold (Au) as PCM and metallic materials, respectively to obtain both broadband ABS and PC^33,37^. Yan et. al. proposed a switchable terahertz metasurface structure with multiple functions based on VO$_{2}$ and Au, which can realize broadband ABS and broadband CPC by treating the insulation to metal phase transition properties of VO$_{2}$^37^. Meanwhile, Song et. al. reported a design of a metasurface utilizing the VO$_{2}$ phase transition from the insulating state to the metallic state to achieve the bi-function of broadband ABS and LPC^33^. However, both RMS structures reported in^33,37^ which using the gold strip for designing of resonant patch for PC mode. Therefore, these structures only efficiently convert the polarization of EM wave with narrow angle tolerance. Furthermore, due to the design of the gold strip is not a diagonal symmetry structure^37^, its polarization conversion ratio (PCR) is strongly dependent on the polarization angle. Inspired by these works, here we propose a RMS structure for both broadband ABS and PC modes using VO$_{2}$ as PCM material as seen in Fig. 2. The top view of resonators in ABS and PC modes is shown in Fig. 2b and c, respectively. To overcome the drawbacks of the gold strip structure for the PC mode, the gradient structure based on a double axes-shaped resonator is utilized and that oriented along diagonally of the unit cell, as seen in Fig. 2c. In this work, we use the Polyimide substrate with dielectric constant of 3.5 and loss tangent of 0.0027. The metallic layers are made of Gold with the conductivity $\sigma$ = 4.56$\times$10$^7$ S/m and the thickness *t* = 200 nm. The unit cell geometrical parameters of the proposed RMS are given by *P* = 40 $\mu$m, $h_1$ = 13.5 $\mu$m, $h_2$ = 16 $\mu$m, *t* = 0.2 $\mu$m, *a* = 8 $\mu$m, *b* = 17 $\mu$m, *r* = 10.5 $\mu$m, *R* = 19 $\mu$m, and $r_c$ = 12.7 $\mu$m, as shown in Fig. 2. The center of circles with radius $r_c$ are O$_1$ (9.9 $\mu$m, −9.9 $\mu$m) and O$_2$ (−9.9 $\mu$m, 9.9 $\mu$m), respectively. It should be noted that our proposed structure has the unit cell dimension of 40 $\mu$m $\times$ 40 $\mu$m $\times$30.3 $\mu$m formed of six thin layers, which is a suitable form for manufacturing with the conventional micro- and nanotechnology. The proposed structure can be patterned by a photolithography, while the thin films of Au and VO$_2$ are deposited on pre-patterned substrate using a conventional sputtering method and the polyimide layers are spin coated. The previously reported work showed that the phase transition of VO$_2$ can be implemented by thermal treatment. The fabrication process of a similar multifunctional terahertz metasurface was also reported in^47^.

The relative permittivity of VO$_{2}$ material is described by Drude model^47–49^:

1 $$\begin{aligned} \varepsilon (\omega ) = \varepsilon _{\infty }-\dfrac{\varepsilon _{p}^2(\sigma )}{\varepsilon ^2+i\gamma \varepsilon } \end{aligned}$$

with epsilon infinity $\varepsilon _{\infty }=12$ and the collision frequency $\gamma =5.75\times 10^{13}$ rad/s^45,48,49^. The plasma frequency can be given by:

2 $$\begin{aligned} \varepsilon _{p}^2(\sigma ) = \dfrac{\sigma }{\sigma _{o}}\varepsilon _{p}^2(\sigma _{o}) \end{aligned}$$

where $\sigma _{o}=3\times 10^5$ S/m and $\varepsilon _{p}^2(\sigma _{o})=1.4\times 10^{15}$ rad/s. It is worthy to note that VO$_{2}$ is a phase transition material that shows the transition behavior from the insulator phase to the metal phase with the increase of temperature above the heating point temperature of 68 °C^45,46^. The conductivity $\sigma$ of VO$_{2}$ material in the insulator phase and metal phase is 200 S/m and 2$\times 10^5$ S/m which corresponds to a resistivity of 0.5 $\Omega$.cm and 0.5 $\times 10^{-3}$ $\Omega$.cm, respectively^41,45,46^.

The simulated results in this work were performed by using the commercial computer simulation technology (CST) Microwave Studio software based on a frequency-domain solver. In this simulation setup, the unit cell boundary conditions are applied to the *x*- and *y*- axis, and the open boundary condition is assigned to the *z*-axis.

In ABS mode, the absorption ($A(\omega )$) of the RMS structure can be determined from the transmittance ($T(\omega )$) and reflectance ($R(\omega )$) with $A(\omega )=S_{11}^2(\omega )$, $T(\omega )=S_{12}^2(\omega )$, and $R(\omega )=S_{21}^2(\omega )$ , as defined in^50^:

3 $$\begin{aligned} A(\omega )=1-R(\omega )-T(\omega ) \end{aligned}$$

Since the metallic layer of VO$_2$ is thicker than the penetration depth of the THz wave, T($\omega$) can be neglected. Therefore, absorptance is determined from the reflectance as $A(\omega )=1-R(\omega )$.

In PC mode, to evaluate the polarization conversion efficiency of the cross-polarization converter, we use a PCR which is defined from the co- and cross-reflection coefficients. When the electric field of incident light is linearly polarized along the *y*-direction ($|E_{iy}|$), PCR is expressed as Eq. 4^13^.

4 $$\begin{aligned} PCR=\frac{|r_{xy}|^2}{|r_{xy}|^2+|r_{yy}|^2} \end{aligned}$$

where the co- and cross-reflection coefficients are defined as $r_{xy}=|E_{rx}|/|E_{iy}|$, and $r_{yy}=|E_{ry}|/|E_{iy}|$ with $|E_{rx}|$ and $|E_{ry}|$ are the magnitude of the electric field of the reflected wave components along *x*- and *y*-axes, respectively.

To further evaluate the performance of the proposed PC, the ellipticity angle ($\eta$) and polarization azimuth angle ($\theta$) for the *y*-polarized wave are calculated from the reflection coefficients. These can be achieved by using the Eqs. 5 and 6 derived from the Stokes parameters^51,52^.

5 $$\begin{aligned} \eta= & {} \frac{1}{2}\arcsin {\left( \frac{2\times p_r\times \sin {(\Delta \varphi })}{1+|p_r|^2} \right) } \end{aligned}$$

6 $$\begin{aligned} \theta= & {} \frac{1}{2}\arctan \left( \frac{2\times p_r\times \cos {(\Delta \varphi })}{1-|p_r|^2} \right) \end{aligned}$$

where $|p_r|=|r_{xy}|/|r_{yy}|$ and $\Delta \varphi =\varphi _{xy}-\varphi _{yy}$. It notes that $\theta$ is the rotation angle between the electric field direction of the reflected wave and the electric field direction of the incident wave. Meanwhile, the polarization state of the reflecting wave is denoted by $\eta$. The reflected wave is linear polarization rotation if $\eta =0^\circ$ and it has another type of polarization rotation if $\eta \ne 0^\circ$. As a result, the *y*-polarization wave can be transformed into its *x*-polarization counterpart if $\eta = 0^o$ and $\theta = \pm 90^o$.

In addition, the absorption and cross-polarization conversion performances are evaluated by the RBW which is calculated as Eq. (7).

7 $$\begin{aligned} RWB = 2\times \dfrac{f_{H}-f_{L}}{f_{H}+f_{L}} \end{aligned}$$

where, $f_H$ and$f_L$ are the highest and lowest working frequency with efficiency over 90%.

## Results and discussion

### The performance and physical mechanism of the proposed RMS

**Figure 3:**
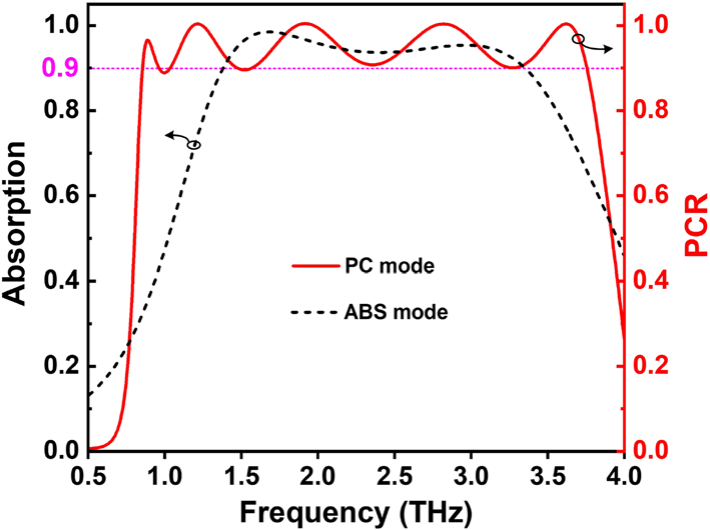
Performance of RMSs for ABS and PC modes.

The proposed RMS is simulated for both metallic and insulator states of VO$_{2}$ corresponding to the working modes of ABS and PC, respectively. Since then its absorption and PCR are calculated as shown in Fig. 3. When VO$_{2}$ is in the fully metallic state with $\sigma$ = 2$\times$10$^5$ S/m, the VO$_{2}$ layer prevents the light transmission thus achieving the perfect absorption as seen in Fig. 3. It can see that the RMS structure exhibits the broadband absorption response with an efficiency higher than 0.9 in the range of 1.36–3.38 THz, corresponding to RBW up to 85$\%$. Two resonant peaks at 1.65 THz and 3.09 THz are observed with efficiency over 0.98 and 0.95, respectively. Meanwhile, when VO$_{2}$ is in the insulator state with $\sigma$ = 200 S/m, the insulator state can transmit the incident wave through the second stack, thus obtaining the cross-polarization conversion as depicted in Fig. 3. It can be found that the PCR of the designed RMS structure can reach above 0.9 in the ultra-wideband frequency of 1.04 - 3.75 THz with the RBW up to 113%. Furthermore, five distinctive resonance peaks at 0.89 THz, 1.22 THz, 1.91 THz, 2.83 THz, and 3.63 THz with intensities of nearly 1 are achieved, which appeared to be the reason for the observed wideband polarization conversion behavior of the designed RMS working in PC mode.

**Figure 4:**
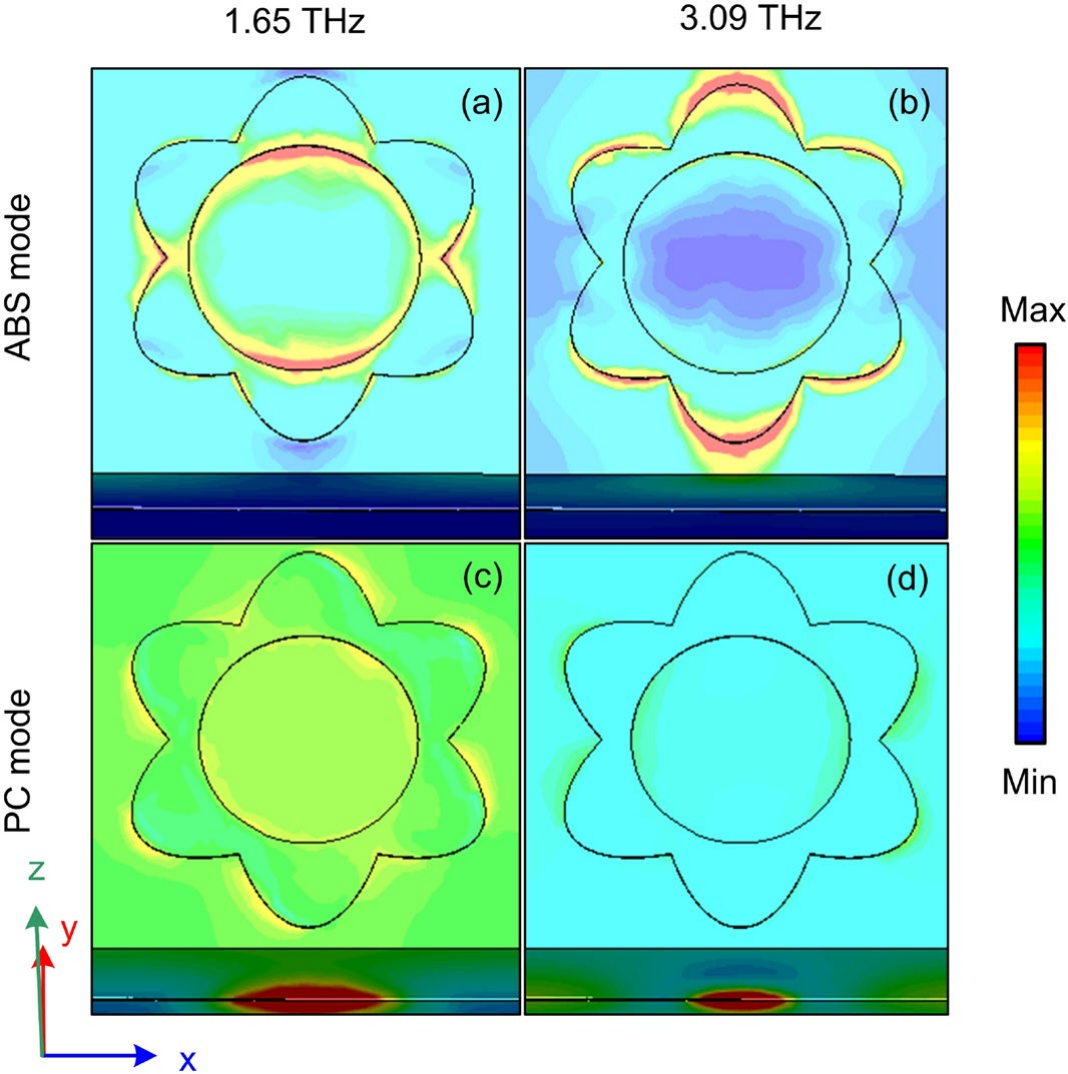
Performance of RMSs for ABS and PC modes.

To understand the physical mechanism behind the switching between the perfect absorption and high efficiency polarization conversion phenomenon, the 3D-view electric field distributions at various frequencies of 1.65 THz and 3.09 THz when the VO$_{2}$ is changed from the metallic state (ABS mode) to insulation state (PC mode) are investigated as shown in Fig. 4. It is clear that when the VO$_{2}$ is in the metallic state, the electric field is only concentrated on the surface of VO$_{2}$ resonator in both frequencies as seen in Fig. 4a and b. Meanwhile, when the VO$_{2}$ is in the insulator state, the electric fields are almost harvested in the middle gold resonator layer (Fig. 4c and d), indicating the gold resonator layer plays a leading role in polarization conversion. This observation proves that the change phase of the middle VO$_{2}$ layer from metallic to insulator phase plays a role in blocking or transmitting the incident EM wave through the device structure, resulting in switching between the perfect ABS and high efficiency PC.

**Figure 5:**
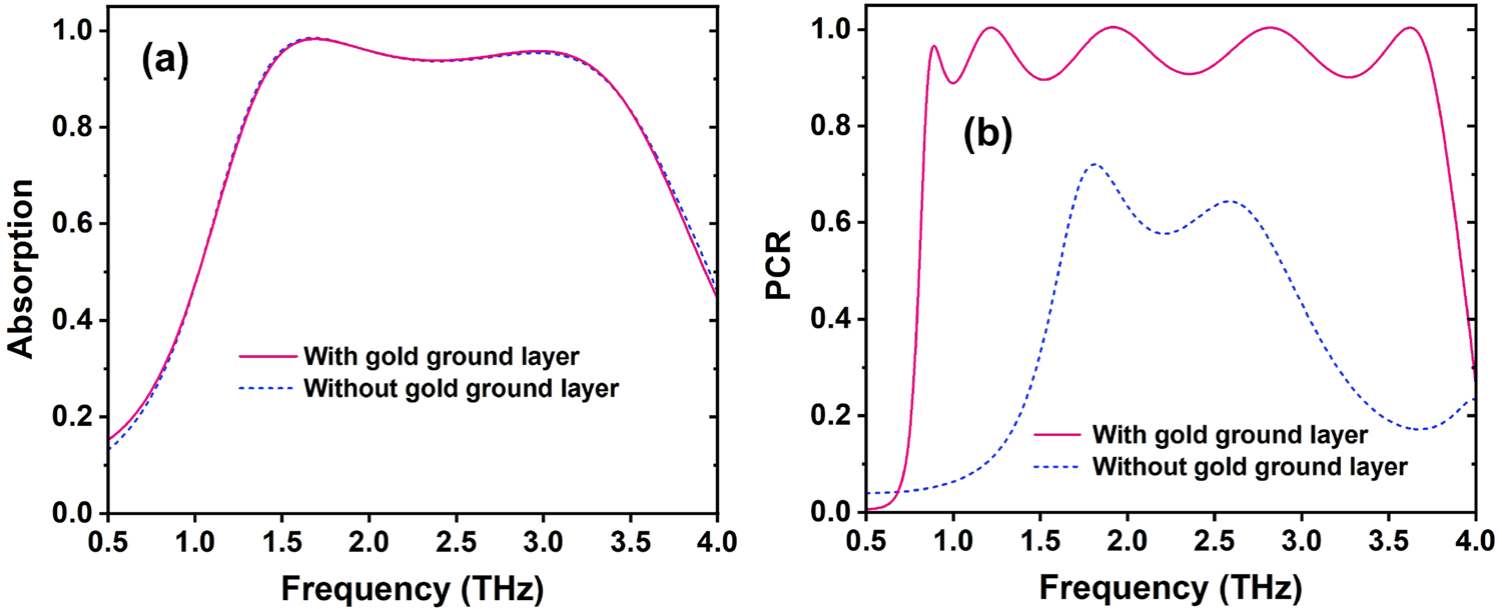
Effect of the continuous gold ground layer on the performance of the proposed RMS for (**a**) ABS and (**b**) PC modes.

To study the effect of the continuous gold ground layer on the performance of the proposed RMS structure for both ABS and PC modes, the ABS and PC spectra of the proposed structure with and without this layer are simulated and the results are illustrated in Fig. 5a and b. As seen in Fig. 5a, the absorption spectrum is not changed when the continuous gold layer is removed, proving that the metallic slab of VO$_2$ can block the EM wave. The EM wave is absorbed in the first stack of a metallic resonator of VO$_2$/dielectric layer/metallic layer of VO$_2$ configuration. Meanwhile, when the continuous gold layer is absent, the PC performance is sharply reduced as depicted in Fig. 5b. It indicates that the continuous gold ground layer plays an important role in blocking the EM transmission, resulting in improving the reflection wave when the proposed RMS works for PC mode.

**Figure 6:**
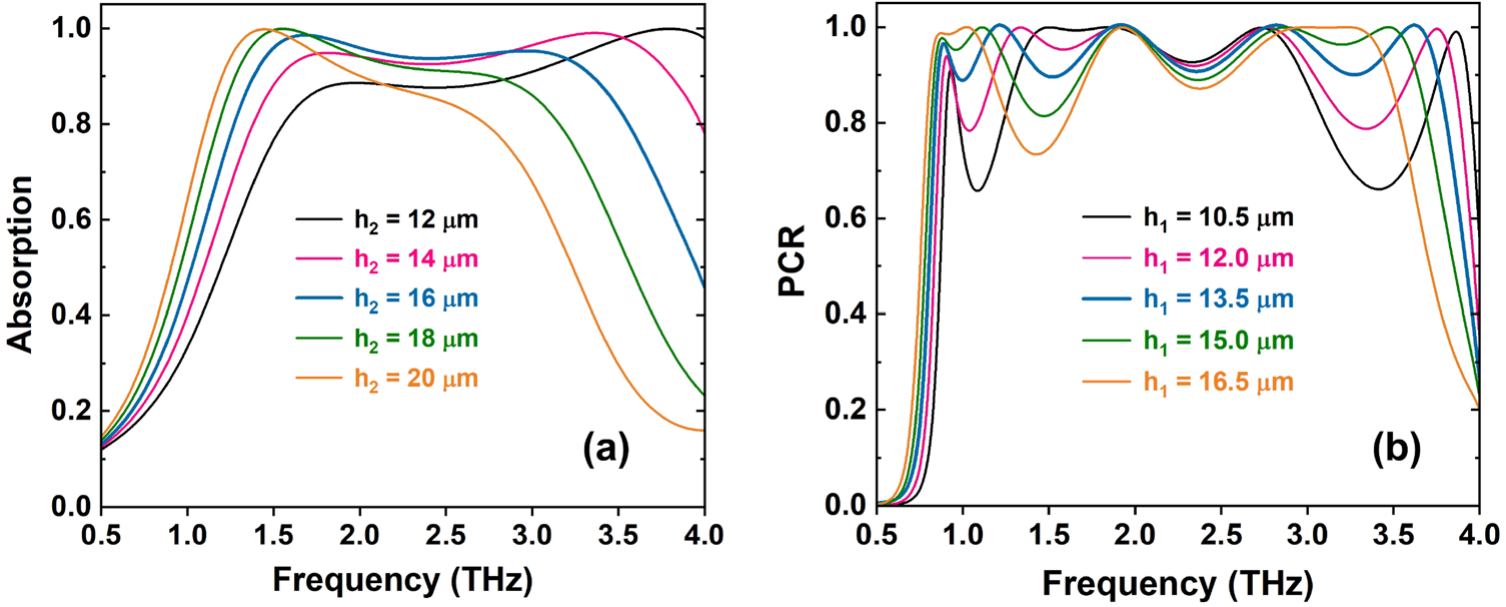
Dependence of the proposed RMS performance on the thickness of dielectric layers: (**a**) $h_2$ and (**b**) $h_1$.

Furthermore, the influence of the thickness of the proposed structure such as the thickness of dielectric layers ($h_2$ and $h_1$) on the performance of the proposed RMS for both ABS and PC modes are also investigated, and the results are shown in Fig. 6. It can see that the change of $h_2$ and $h_1$ values affect the ABS and PC performances of the proposed RMS, respectively. With changing of $h_2$ value, both absorption intensity and absorption spectrum are varied as depicted in Fig. 6a. Furthermore, with increasing of $h_2$ value, the absorption spectrum is red shifted which corresponds to the increase in the effective optical path for ABS mode. The optimized value of $h_2$ is chosen at 16 $\mu$m for obtaining both the highest absorptivity and widest bandwidth. Similar trend with the variation of $h_2$, when the $h_1$ value is changed, both the PC efficiency and frequency band is varied (Fig. 6b). With the $h_1$ value of 13.5 $\mu$m, optimized PC performance is obtained.

To further reveal the characteristics and physical mechanism of proposed RMS structure, parametric studies in both functions of absorption and polarization conversion are discussed in next sections.

### Absorption characterization

**Figure 7:**
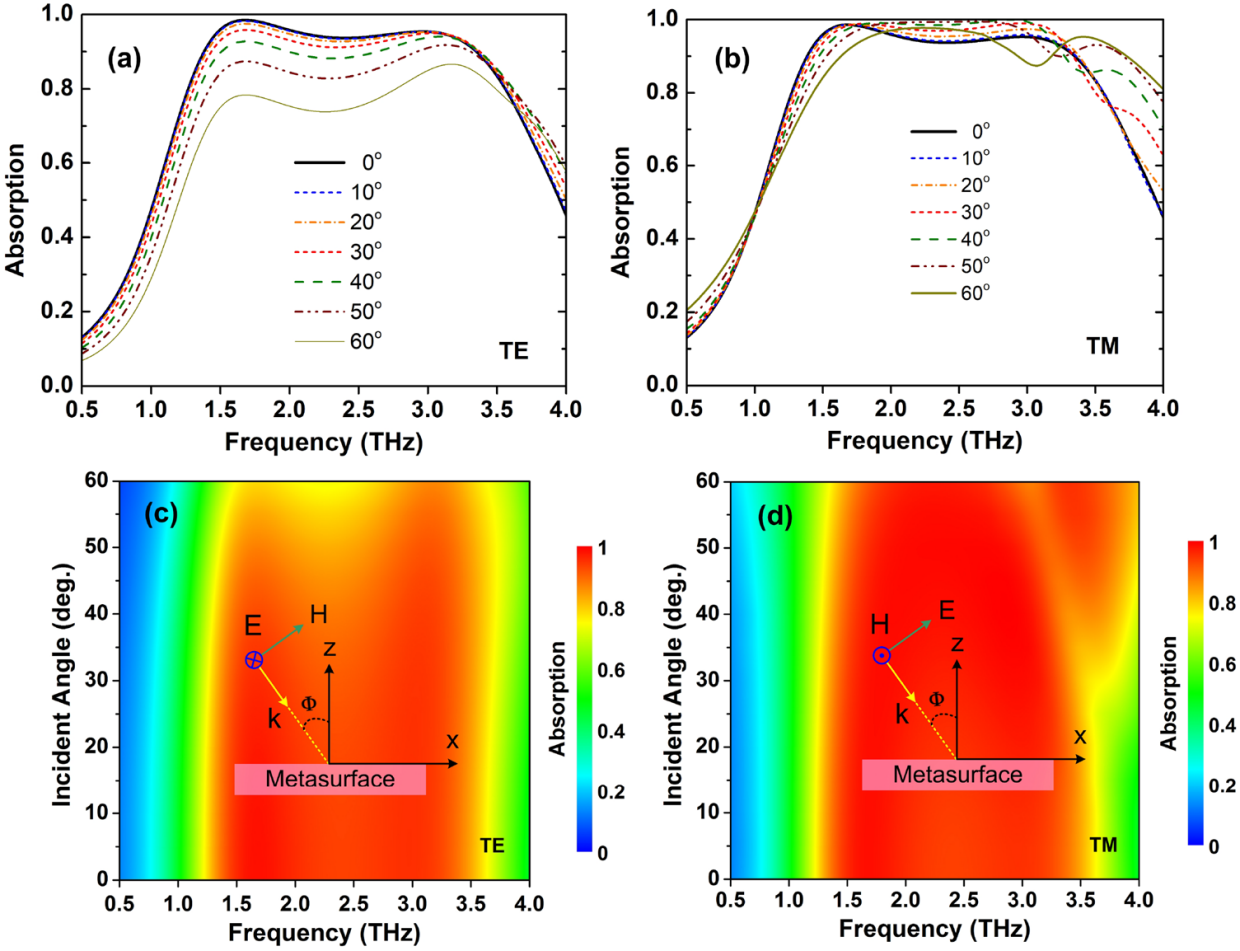
Dependence of absorption on incident angle ($\Phi$) of the proposed RMS for ABS mode under (**a, c**) TE and (**b, d**) TM polarizations.

Incident and polarization angle insensitivity are critical properties for practical applications of perfect absorbers because of the variety of incident EM waves. Fig. 7 shows the absorptivity plots with different incident angles ranging from 0 to 60° under TE and TM polarizations, respectively. As seen in Fig. 7, the designed structure exhibits high absorptivity in a wide incident angle for both TE and TM polarizations. In TE polarization, the absorptivity is decreased with increasing the incident angle, and absorptivity is still kept higher than 0.8 for incident angle up to 50°. In TM polarization, it is interesting to observe that the absorptivity increases with increasing incident angle. Furthermore, the absorption spectrum is expanded to the high frequencies when the angle of incidence is greater than 30$^o$. The same phenomenon is reported in previous work^26^.

**Figure 8:**
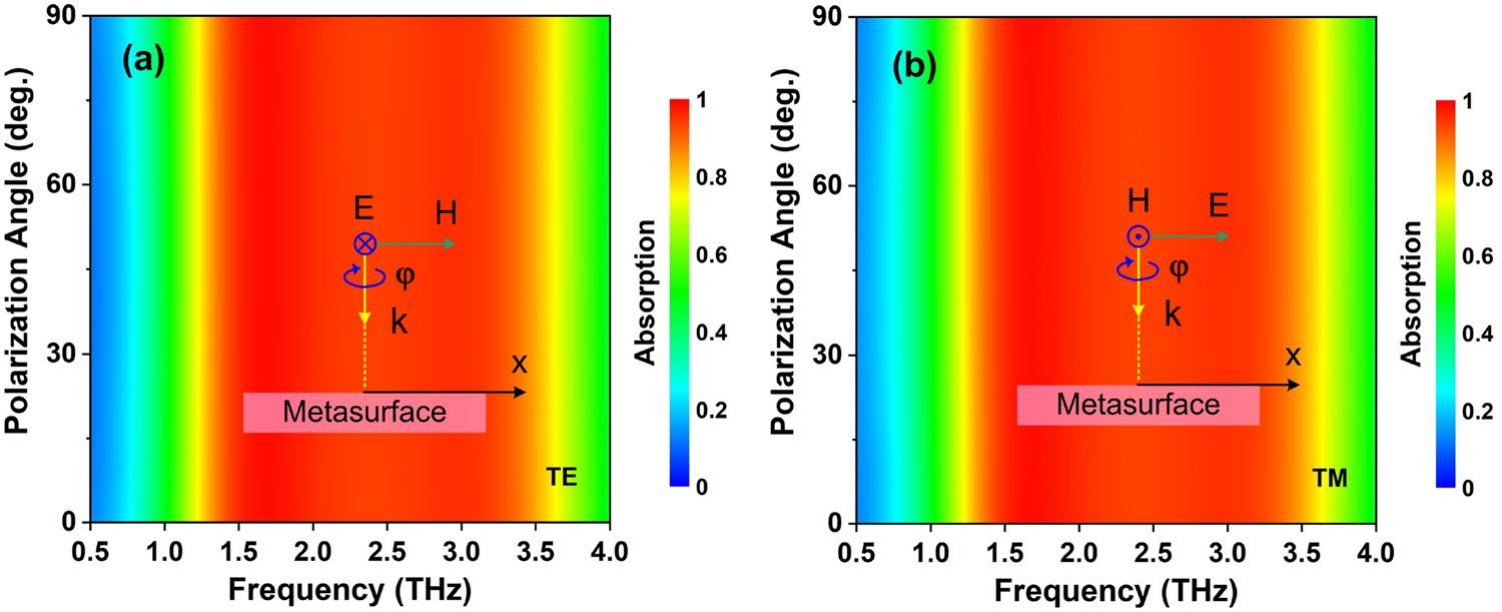
Dependence of absorption on polarization angle ($\varphi$) of the proposed RMS for ABS mode under (**a**) TE and (**b**) TM polarizations.

The absorptivities for different polarization angles in both TE and TM polarizations are simulated, and the results are presented in Fig. 8. The absorptivity does not change with polarization angle variation from 0 to 90° for both TE and TM polarizations (Fig. 8). It indicates this structure is insensitive to all polarization angles due to the symmetric structure of the metallic VO$_{2}$ resonator.

The impedance matching between the proposed structure and free space is used to explain the wideband absorption mechanism. The normalized impedance of the designed structure is calculated by Eq. (8)^11,53,54^.

8 $$\begin{aligned} Z = \sqrt{\dfrac{(1+S_{11})^2-S_{21}^2}{(1-S_{11})^2-S_{21}^2}}=\dfrac{1+S_{11}}{1-S_{11}} \end{aligned}$$

**Figure 9:**
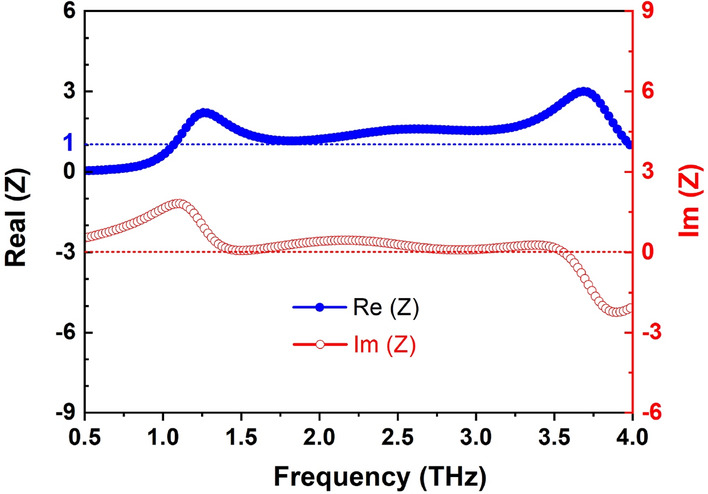
Normalized impedance of the proposed RMS for ABS mode.

As depicted in Fig. 9, the real component of the input normalized impedance is nearly 1, while the imaginary component is approximately equal to 0 in the wide frequency range from 1.65 to 3.09 THz. It indicates that the wideband impedance matching is achieved, resulting in the realization of the wideband absorption response.

**Figure 10:**
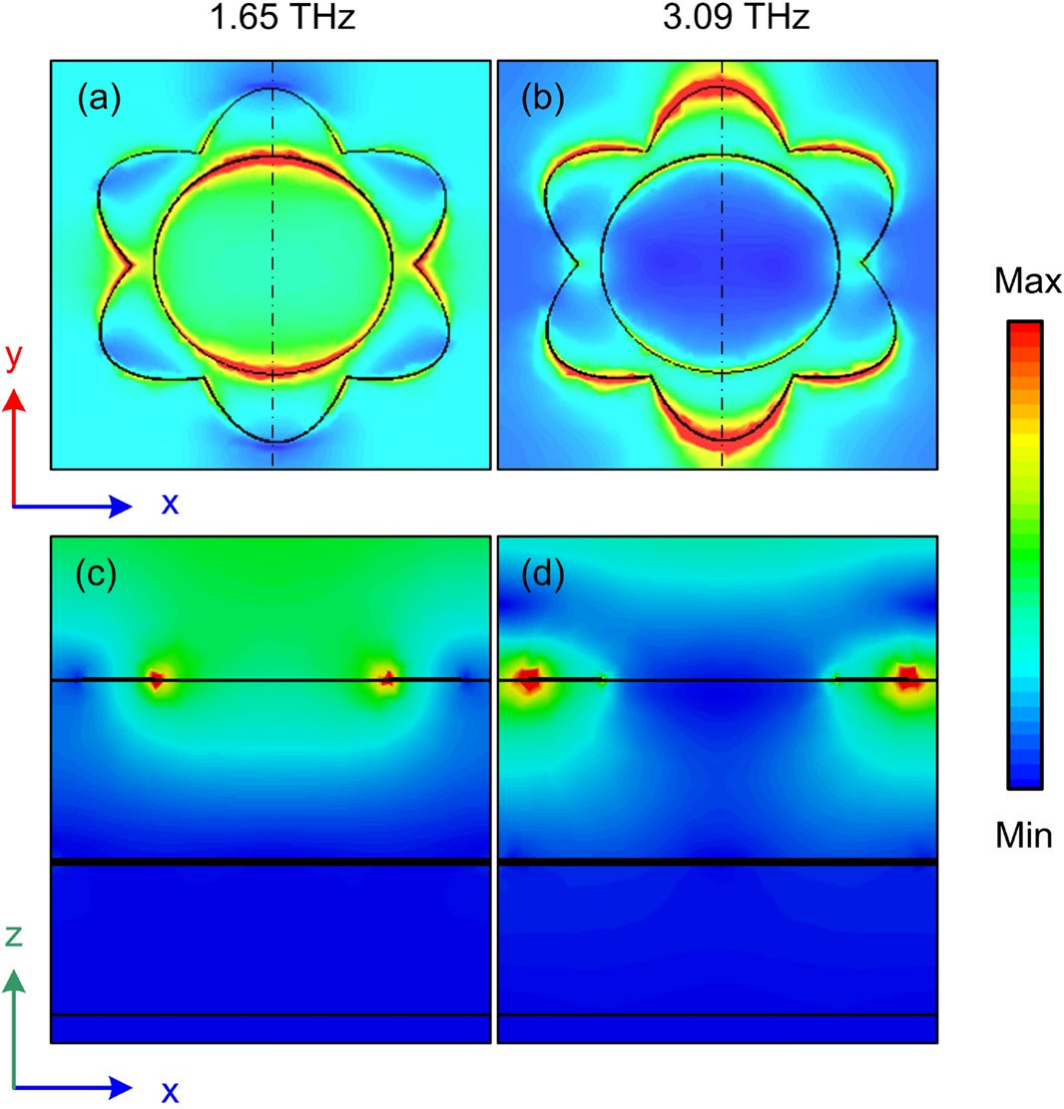
Electric field distribution in a unit cell for *y*-polarized incident waves at the resonant frequencies in (**a**) XY plane (top-view) and (**b**) XZ plane (determined at the position by the black dash-dotted lines in Fig. 10.

To gain an insight into the absorption mechanism, we have investigated the distribution of electric fields at two resonant frequencies of 1.96 THz and 3.09 THz under normal incidence, gathered in the XY and XZ planes as seen in Fig. 10. The electric field is mainly concentrated on two sides of the inner ring of a flower-shaped resonator (Fig. 10a and c), and this electric field is localized inside the ring, forming this resonance from the corresponding electric dipole mode of the inner ring at 1.96 THz. Meanwhile, the electric field distribution is accumulating at the flower-shaped resonator’s outer petals at a higher frequency of 3.09 THz (Fig. 10b). Moreover, this field is strongly coupled to the petals of neighboring unit cells, so the resonance is due to the electric dipoles that occur in the petals between the neighboring unit cells. It was reported that higher lossy metals such as Cr or VO$_2$ can decrease the quality factor of dipole resonances and thus increase the absorption bandwidth when compared to other noble metals like Au and Ag^41,55^.

### Polarization conversion characterization

**Figure 11:**
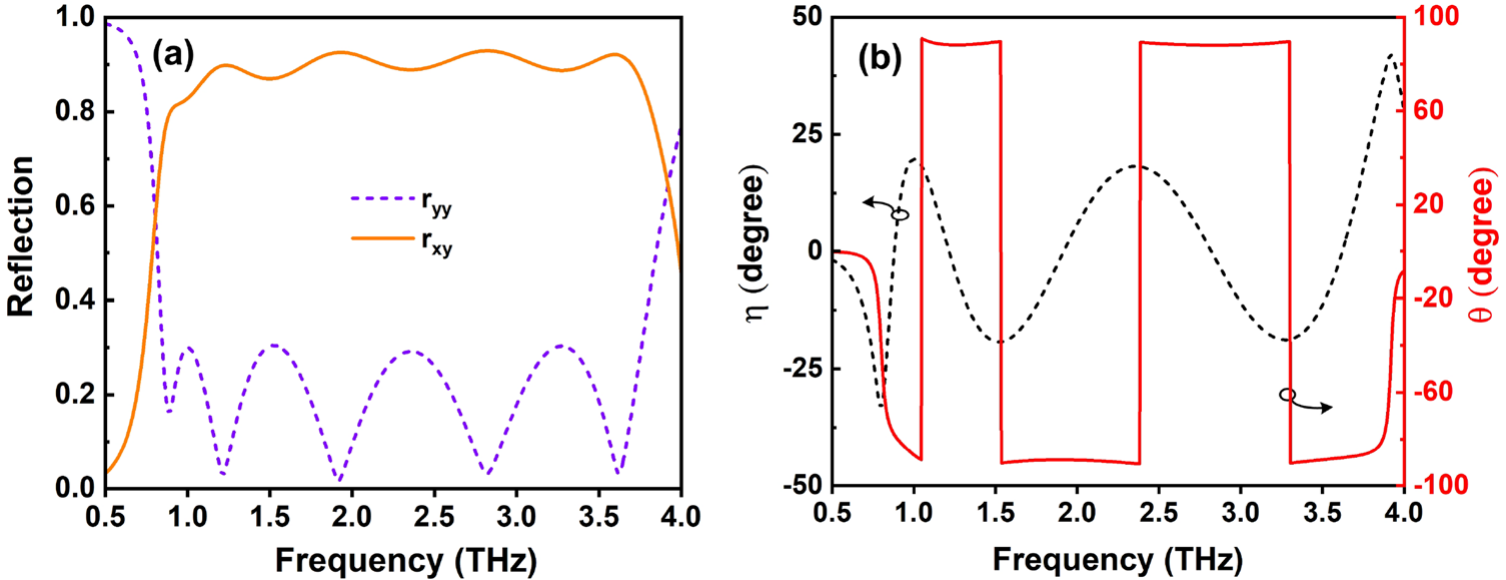
(**a**) The simulated magnitude of reflection coefficients and (**b**) the calculated ellipticity angle ($\eta$) and polarization azimuth angle ($\theta$) of the proposed RMS for PC mode.

To better understand the polarization state of an EM wave, ellipticity ($\eta$) and polarization azimuth angle ($\theta$) for the y-polarized under normal incidence are investigated, and the results are shown in Fig. 11. From 1.04 to 3.75 THz, the ellipticity of the incident y-polarized wave is less than 20$^o$, while the polarization azimuth angle is nearly 90$^o$, confirming that linear polarization conversion is achieved throughout the operating band.

**Figure 12:**
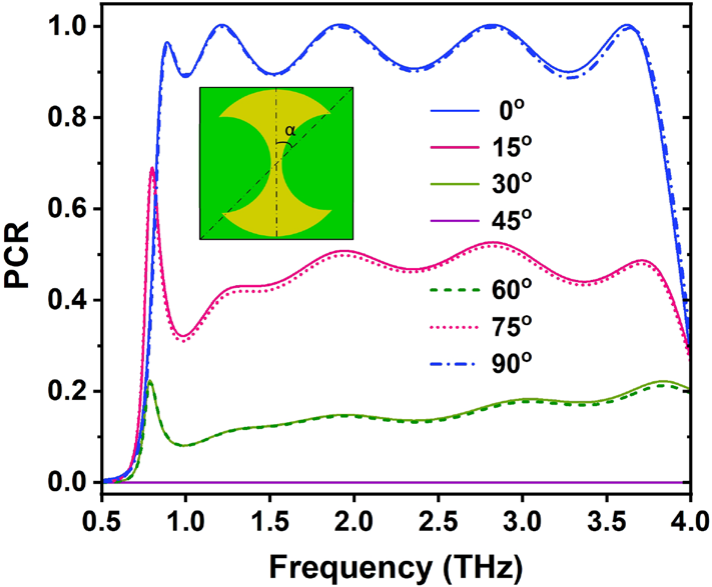
Dependence of PCR on rotation angle ($\alpha$) under normal incidence. The rotation angle ($\alpha$) is the angle between the long base line of the gold resonant patch and the diagonally of the unit cell.

To study the effect of the orientation of the gold resonant patch on the PC performance, we have investigated the PCR spectra of the proposed RMS with various rotation angles ($\alpha$) under normal incidence, and the results are presented in Fig. 12. Due to its asymmetric structure, the proposed RMS shows different PCR level for the TE and TM polarizations when the rotation angle is changed, as shown in Fig. 12. The PC efficiency decreases as the rotation angle increases from 0 to 45°, then increases as the rotation angle increases from 45 to 90°. Furthermore, the PC efficiency remains nearly constant for rotation angles of 0° and 90°, 15° and 75°, and 30° and 60°, respectively. However, these bandwidth remains nearly constant as the rotation angle varies. The obtained results suggest that the highest PC performance can be obtained by designing of the resonant patch of polarization converter along diagonally of the unit cell.

**Figure 13:**
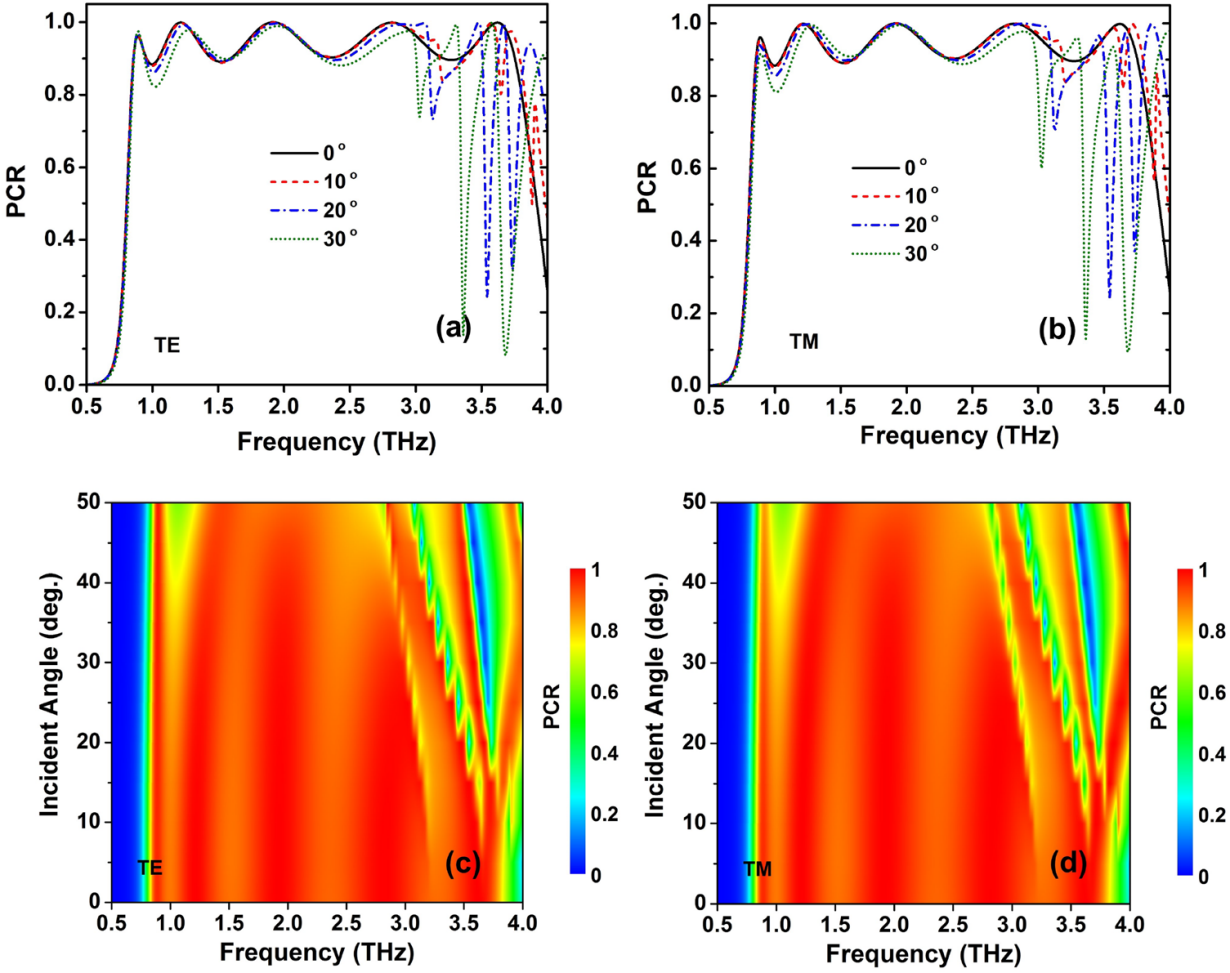
Dependence of PCR on incident angle of the proposed RMS for PC mode: (**a, c**) TE mode and (**s, d**) TM mode. In (**a** and **b**) plot the PCR spectra at the selected incident angles.

Figure 13 depicts the effect of the variation of the incidence angle on the PCR spectrum under both TE and TM polarizations. The designed structure shows a stable efficiency with high incident angle tolerance in the large incident angle from 0 to 50° in both TE and TM polarizations as seen in Fig. 13. Meanwhile, the bandwidth becomes narrower in the higher frequency band as the incidence angle increases. This can be explained mainly due to the destructive interference between the reflected waves at the metasurface^56^. However, the PCR is still higher than 0.8 in the frequency range of 1.04–3.0 THz when the incident angle increases up to 40°, indicating that the designed structure achieved good PC characteristics even at wide incidence angles.

**Figure 14:**
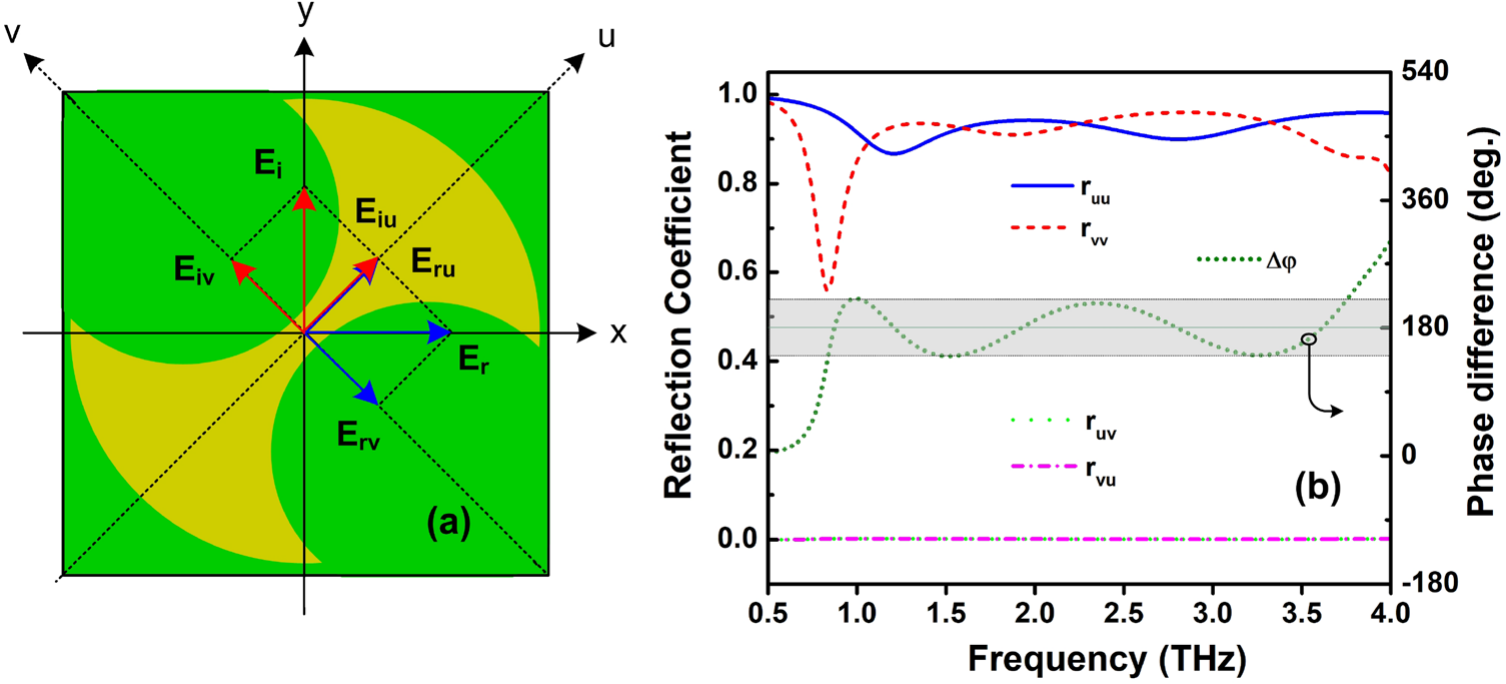
(**a**) Working principle of the proposed RMS for PC mode and (**b**) the magnitude of the reflection coefficients and their different phase of *u*- and *v*-components.

To study the working principle of the proposed RMS in PC mode, we have analyzed its response in the *uv*-coordinate system, as presented in Fig. 14a. The *y*-polarized incident EM wave ($E_i$) can be decomposed into two orthogonal part of $E_{iu}$ and $E_{iv}$ in the *uv*-coordinate system. The incident and reflection waves are expressed by Eqs. (9) and (10)^57^.

9 $$\begin{aligned} E_i=\; & {} \hat{y}E_i=\hat{u}E_{iu}+\hat{v}E_{iv} \end{aligned}$$

10 $$\begin{aligned} E_r= \;& {} \hat{u}E_{ru}+\hat{v}E_{rv}=\hat{u}(r_{uu}E_{iu}e^{i\varphi _{uu}}+r_{uv}E_{iv}e^{i\varphi _{uv}})+\hat{v}(r_{vv}E_{iv}e^{i\varphi _{vv}}+r_{vu}E_{iu}e^{i\varphi _{vu}}) \end{aligned}$$

where, $\hat{v}$ and $\hat{u}$ are unit vectors; $r_{uu}$ and $\varphi _{uu}$, $r_{vv}$ and $\varphi _{vv}$, $r_{uv}$ and $\varphi _{uv}$, $r_{vu}$ and $\varphi _{vu}$ are magnitude and phase of co- and cross-reflections in the *uv*-coordinate system, respectively. Due to the asymmetric shape of the proposed structure for PC mode, the magnitude and phase of reflection waves in the *u*- and *v*- directions are different. As seen in Fig. 14a, the synthesis of $E_{ru}$ and $E_{rv}$ will orient along the *x*-axis if $r_{uu} = r_{vv}$ = 1, $r_{uv} = r_{vu}$ = 0, and $\Delta \varphi = |\varphi _{uu} - \varphi _{vv}| = \pi +2k\pi$. Therefore, the co- and cross-polarization reflection coefficients and the phase difference of co-polarization reflection response versus frequency are investigated as shown in Fig. 14b. It is clear that the magnitude of co- and cross-polarization reflections are nearly equal to 0 and 1, respectively at the whole band, while the phase difference floats around 180 ± 40° and is equal to 180° at the exact five resonant frequencies as mentioned in Fig. 11, proving that the designed structure reveals the high efficiency and broadband cross-polarization conversion characteristics.

**Figure 15:**
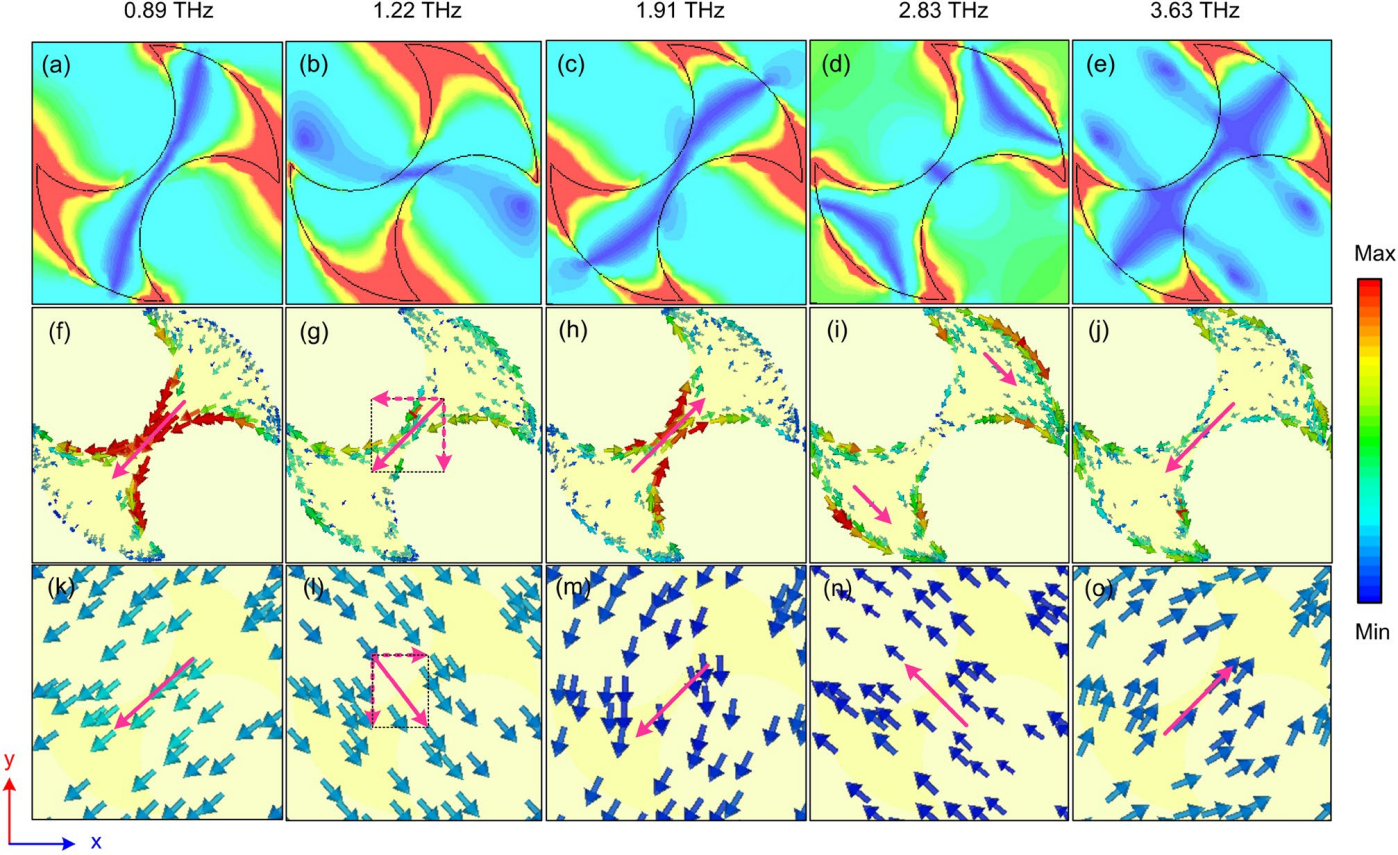
The distributes of (**a–e**) electric field, (**f–j**) the top surface current, and (**k–o**) the bottom surface current of the proposed RMS for PC mode at various resonant frequencies.

The origin of the physical mechanism of polarization conversion is an important issue, whether it is caused by an electric and/or magnetic resonance. To gain insight into the physics mechanism of the designed structure, the electric field and the surface current distributions on the RMS structure for PC mode are simulated at the frequencies according to the five resonance peaks. From Fig. 15a–e, the electric field is mainly localized in the edges of the axes of the designed structure. Furthermore, at a specified frequency, the electric field is concentrated on a certain part of the RMS structure. The top and bottom surface current distributions at five frequencies are illustrated in Fig. 15f–o. At higher resonance frequencies of 1.91 THz, 2.83 THz, and 3.63 THz, the top surface current is anti-parallel with the bottom surface current, which indicates that strong magnetic resonance contributes to these resonant frequencies. Meanwhile, at the lowest frequency of 0.89 GHz, the top surface current is parallel with the bottom current, so this resonance frequency is due to electric resonance. At a resonant frequency of 1.22 THz, however, the synthesis of top and bottom surface currents is parallel and anti-parallel in the *x* and *y* axes, respectively. Therefore, this resonant frequency is fully understood to be magnetic and electric resonances. Based on the observed results, it can be concluded that the wideband polarization conversion mechanism is due to a combination of multi-resonances generated by both electric and magnetic resonances.

Finally, the performance comparison of our structure with the existing state-of-the-art RMSs in the THz range is summarized in Table 1, indicating that the proposed structure has greatly improved features in terms of efficiency and bandwidth.

**Table 1:** Comparison with the existing state-of-the-art RMSs in THz range.

Ref.	Functionality	Working band (THz)	Efficiency ($\%$)	RBW ($\%$)	Active materials
^26^	ABS	1.54–4.54	>80	96	Graphene
	LPC	2.11–3.63	>90	53	
^33^	ABS	0.52–1.2	>90	79	VO$_2$
	LPC	0.42–10.4	>90	85	
^34^	ABS	6.3–15.0	>90	82	VO$_2$
	CPC	10.8-14.4	>90	57	
^37^	ABS	0.74–1.62	>90	75	VO$_2$
	CPC	1.47–2.27	>70	86	
This work	ABS	1.36–3.38	>90	85	VO$_2$
	LPC	1.04–3.75	>90	113	

## Conclusion

We proposed a reconfigurable broadband terahertz metasurface based on the VO$_{2}$ phase change material, which achieved two functions with near perfect absorption and high efficiency reflective cross-polarization conversions. When VO$_{2}$ is in the metallic state, the proposed metasurface exhibited nearly perfect absorption in the wideband in the range of 1.36–3.38 THz with relative bandwidth up to 85 $\%$. Once VO$_{2}$ is in the insulator state, the cross-polarization conversion response can be obtained with the linear polarization conversion ratio exceeds 90$\%$ in the wideband of 1.04–3.75 THz with the corresponding relative bandwidth up to 113 $\%$. The physical mechanism of the cross-polarization conversion effect can be attributed to the multi-mode of magnetic and electric resonances. Besides the excellent performance at normal incidence, the broadband and high-efficiency performances of both absorption and linear polarization conversion of the proposed structure were maintained for a wide incidence angle. Finally, compared with the existing state-of-the-art multi-functional metasurface, the performance of the designed reconfigurable metasurface showed superior structure in terms of high efficiency and wideband. Our proposed reconfigurable structure may provide a new approach to designing high-performance and enabling emergent metasurface functionalities for applications in the technologically difficult terahertz-frequency regime.

## Data Availability

All data generated or analysed during this study are included in this published article (and its Supplementary Information files).

## References

[1] Smith DR, Padilla WJ, Vier DC, Nemat-Nasser SC, Schultz S (2000). Composite medium with simultaneously negative permeability and permittivity. Phys. Rev. Lett..

[2] Schurig D (2006). Metamaterial electromagnetic cloak at microwave frequencies. Science.

[3] Pendry JB (2000). Negative refraction makes a perfect lens. Phys. Rev. Lett..

[4] Bukhari SS, Vardaxoglou JY, Whittow W (2019). A metasurfaces review: Definitions and applications. Appl. Sci..

[5] Wang HL, Ma HF, Chen M, Sun S, Cui TJ (2021). A reconfigurable multifunctional metasurface for full-space control of electromagnetic waves. Adv. Funct. Mater..

[6] Yu N (2011). Light propagation with phase discontinuities: Generalized laws of reflection and refraction. Science.

[7] Ni X, Emani NK, Kildishev AV, Boltasseva A, Shalaev VM (2012). Broadband light bending with plasmonic nanoantennas. Science.

[8] Aieta F (2012). Aberration-free ultrathin flat lenses and axicons at telecom wavelengths based on plasmonic metasurfaces. Nano Lett..

[9] Chen WT (2014). High-efficiency broadband meta-hologram with polarization-controlled dual images. Nano Lett..

[10] Guo W, Liu Y, Han T (2016). Ultra-broadband infrared metasurface absorber. Opt. Express.

[11] Nguyen TQM (2021). Numerical study of an ultra-broadband and wide-angle insensitive perfect metamaterial absorber in the uv-nir region. Plasmonics.

[12] Hao J (2007). Manipulating electromagnetic wave polarizations by anisotropic metamaterials. Phys. Rev. Lett..

[13] Nguyen TQH (2021). Simple design of a wideband and wide-angle reflective linear polarization converter based on crescent-shaped metamaterial for ku-band applications. Opt. Commun..

[14] Peralta XG (2009). Metamaterials for thz polarimetric devices. Opt. Express.

[15] Wu PC (2017). Broadband wide-angle multifunctional polarization converter via liquid-metal-based metasurface. Adv. Opt. Mater..

[16] Liu C (2019). High-performance bifunctional polarization switch chiral metamaterials by inverse design method. npj Comput. Mater..

[17] Nguyen TKT (2021). Simple design of efficient broadband multifunctional polarization converter for x-band applications. Sci. Rep..

[18] Barkabian M, Sharifi N, Granpayeh N (2021). Multi-functional high-efficiency reflective polarization converter based on an ultra-thin graphene metasurface in the thz band. Opt. Express.

[19] Wang J, Yang R, Li Z, Tian J (2022). Reconfigurable multifunctional polarization converter based on asymmetric hybridized metasurfaces. Opt. Mater..

[20] Tang B, Ren Y (2022). Tunable and switchable multi-functional terahertz metamaterials based on a hybrid vanadium dioxide-graphene integrated configuration. Phys. Chem. Chem. Phys..

[21] Li X (2019). Switchable multifunctional terahertz metasurfaces employing vanadium dioxide. Sci. Rep..

[22] Gao Z (2021). Multifunctional ultra-thin metasurface with low infrared emissivity, microwave absorption and high optical transmission. Opt. Commun..

[23] Ren Y, Tang B (2021). Switchable multi-functional vo2-integrated metamaterial devices in the terahertz region. J. Lightwave Technol..

[24] Wang D, Sun S, Feng Z, Tan W (2020). Enabling switchable and multifunctional terahertz metasurfaces with phase-change material. Opt. Mater. Express.

[25] Cheng H (2017). Integrating polarization conversion and nearly perfect absorption with multifunctional metasurfaces. Appl. Phys. Lett..

[26] Peng L, Jiang X, Li S-M (2018). Multi-functional device with switchable functions of absorption and polarization conversion at terahertz range. Nanoscale Res. Lett..

[27] Mao M (2019). Dynamically temperature-voltage controlled multifunctional device based on vo2 and graphene hybrid metamaterials: Perfect absorber and highly efficient polarization converter. Nanomaterials.

[28] Zhou Y (2018). A multifunctional metasurface with integrated absorption and polarization rotation. Mater. Res. Express.

[29] Dutta R, Mitra D, Ghosh J (2020). Dual-band multifunctional metasurface for absorption and polarization conversion. Int. J. RF Microwave Comput.-Aided Eng..

[30] Ha DT (2021). Switching between perfect absorption and polarization conversion, based on hybrid metamaterial in the GHz and THz bands. J. Phys. D: Appl. Phys..

[31] Liu Y, Huang X, Yang H, Hua L, Lei Y (2020). Zigzag reflective multifunctional metamaterial absorber and polarization rotator with horizontal strip structure. Phys. Scr..

[32] Li Z (2021). Multifunctional metasurface for broadband absorption, linear and circular polarization conversions. Opt. Mater. Express.

[33] Song Z, Zhang J (2020). Achieving broadband absorption and polarization conversion with a vanadium dioxide metasurface in the same terahertz frequencies. Opt. Express.

[34] He J (2022). Lightweight switchable bifunctional metasurface based on vo2: High-efficiency absorption and ultra-wideband circular polarization conversion. Optik.

[35] Lv F, Xiao Z, Lu X, Chen M, Zhou Y (2021). Polarization conversion and absorption of multifunctional all-dielectric metamaterial based on vanadium dioxide. Plasmonics.

[36] Qiu Y (2022). Vanadium dioxide-assisted switchable multifunctional metamaterial structure. Opt. Express.

[37] Yan D, Meng M, Li J, Li J, Li X (2020). Vanadium dioxide-assisted broadband absorption and linear-to-circular polarization conversion based on a single metasurface design for the terahertz wave. Opt. Express.

[38] Cheng J, Li J (2022). Switchable terahertz broadband absorption and linear-to-circular polarization conversion. J. Modern Opt..

[39] Le DH, Lim S (2019). Four-mode programmable metamaterial using ternary foldable origami. ACS Appl. Mater. Interfaces.

[40] Li Y (2020). A tunable metasurface with switchable functionalities: From perfect transparency to perfect absorption. Adv. Opt. Mater..

[41] Ding F, Zhong S, Bozhevolnyi SI (2018). Vanadium dioxide integrated metasurfaces with switchable functionalities at terahertz frequencies. Adv. Opt. Mater..

[42] Wang Q (2016). Optically reconfigurable metasurfaces and photonic devices based on phase change materials. Nature Photonic.

[43] Masyukov M (2020). Photo-tunable terahertz absorber based on intercalated few-layer graphene. J. Opt..

[44] Huang J (2020). Active controllable dual broadband terahertz absorber based on hybrid metamaterials with vanadium dioxide. Opt. Express.

[45] Liu M (2012). Terahertz-field-induced insulator-to-metal transition in vanadium dioxide metamaterial. Nature.

[46] Zhang H-T (2015). Wafer-scale growth of vo2 thin films using a combinatorial approach. Nat. Commun..

[47] Jepsen PU (2006). Metal-insulator phase transition in a $${\rm V}{{\rm o}}_{2}$$ thin film observed with terahertz spectroscopy. Phys. Rev. B.

[48] Mou N, Tang B, Li J, Dong H, Zhang L (2022). Switchable ultra-broadband terahertz wave absorption with vo2-based metasurface. Sci. Rep..

[49] Ge J, Zhang Y, Dong H, Zhang L (2022). Nanolayered vo2-based switchable terahertz metasurfaces as near-perfect absorbers and antireflection coatings. ACS Appl. Nano Mater..

[50] Landy NI, Sajuyigbe S, Mock JJ, Smith DR, Padilla WJ (2008). Perfect metamaterial absorber. Phys. Rev. Lett..

[51] Shi H, Li J, Zhang A, Wang J, Xu Z (2014). Broadband cross polarization converter using plasmon hybridizations in a ring/disk cavity. Opt. Express.

[52] Xiao Z, Zou H, Zheng X, Ling X, Wang L (2017). A tunable reflective polarization converter based on hybrid metamaterial. Opt. Quant Electron.

[53] Smith DR, Vier DC, Koschny T, Soukoulis CM (2005). Electromagnetic parameter retrieval from inhomogeneous metamaterials. Phys. Rev. E.

[54] Tuan TS, Lam VD, Hoa NTQ (2019). Simple design of a copolarization wideband metamaterial absorber for c-band applications. J. Electron. Mater..

[55] Zhu J (2014). Ultra-broadband terahertz metamaterial absorber. Appl. Phys. Lett..

[56] Xu J, Li R, Qin J, Wang S, Han T (2018). Ultra-broadband wide-angle linear polarization converter based on h-shaped metasurface. Opt. Express.

[57] Cao TN, Nguyen MT, Nguyen NH, Truong CL, Nguyen TQH (2021). Numerical design of a high efficiency and ultra-broadband terahertz cross-polarization converter. Mater. Res. Express.

